# Generation of Variants in *Listeria monocytogenes* Continuous-Flow Biofilms Is Dependent on Radical-Induced DNA Damage and RecA-Mediated Repair

**DOI:** 10.1371/journal.pone.0028590

**Published:** 2011-12-05

**Authors:** Stijn van der Veen, Tjakko Abee

**Affiliations:** 1 Top Institute Food and Nutrition (TIFN), Wageningen, The Netherlands; 2 Laboratory of Food Microbiology, Wageningen University and Research Centre, Wageningen, The Netherlands; Auburn University, United States of America

## Abstract

The food-borne pathogen *Listeria monocytogenes* is a Gram-positive microaerophilic facultative anaerobic rod and the causative agent of the devastating disease listeriosis. *L. monocytogenes* is able to form biofilms in the food processing environment. Since biofilms are generally hard to eradicate, they can function as a source for food contamination. In several occasions biofilms have been identified as a source for genetic variability, which potentially can result in adaptation of strains to food processing or clinical conditions. However, nothing is known about mutagenesis in *L. monocytogenes* biofilms and the possible mechanisms involved. In this study, we showed that the generation of genetic variants was specifically induced in continuous-flow biofilms of *L. monocytogenes*, but not in static biofilms. Using specific dyes and radical inhibitors, we showed that the formation of superoxide and hydroxyl radicals was induced in continuous-flow biofilms, which was accompanied with in an increase in DNA damage. Promoter reporter studies showed that *recA*, which is an important component in DNA repair and the activator of the SOS response, is activated in continuous-flow biofilms and that activation was dependent on radical-induced DNA damage. Furthermore, continuous-flow biofilm experiments using an in-frame *recA* deletion mutant verified that RecA is required for induced generation of genetic variants. Therefore, we can conclude that generation of genetic variants in *L. monocytogenes* continuous-flow biofilms results from radical-induced DNA damage and RecA-mediated mutagenic repair of the damaged DNA.

## Introduction

*Listeria monocytogenes* is a food-borne pathogen and the cause of listeriosis, which is a disease that is associated with meningitis, encephalitis, or spontaneous abortions [1]. In 99% of the cases listeriosis is the result of consumption of contaminated food products [2]. *L. monocytogenes* is frequently encountered in food processing facilities, where it thrives in the form of biofilms on food processing equipment or in pipelines [3], [4]. Biofilms, which are defined as structured communities of microorganisms that are attached to a surface, are generally more resistant to antimicrobial agents and disinfectants than planktonic cells and can therefore act as a source of food contamination [5]. Previously, it has been suggested that bacterial cells present in the different microniches of biofilms experience various stresses and hence activate stress resistance mechanisms [6]. For *L. monocytogenes* biofilms, it has indeed been shown that various stress mechanisms are activated in different types of biofilms and that some of them are involved in the increased resistance of biofilms against disinfectants [7], [8], [9]. Thus far, most of the studies on *L. monocytogenes* biofilm formation focus on static conditions. Static biofilms of *L. monocytogenes* consist of small rod-shaped cells that are attached as microcolonies or homogeneous layers [10], [11]. However, *L. monocytogenes* biofilms formed under continuous flowing conditions appeared to consist of a dense network of knitted-chains that are composed of elongated cells and surround ball-shaped microcolonies [12]. This type of biofilm is encountered in for instance industrial pipelines of food processing facilities. A major outbreak of *L. monocytogenes* has previously been related with contaminated chocolate milk tank draining pipes [13]. Furthermore, *L. monocytogenes* has been encountered in whey transport pipes [14]. Recently, we showed that the formation of continuous-flow biofilms is dependent on the activation of the SOS response factor YneA, which is involved in the formation of elongated cells [7].

One of the important phenomena that has been attributed to bacterial biofilms is the generation of genetic variants that might result in the persistence of adapted strains in the industry or in human infections [15], [16]. While the mechanism for the generation of variants in different types of biofilms for most organisms is not exactly known, biofilms have been described to be responsible for diversity within various bacterial populations [17]. For the pathogens *Streptococcus pneumoniae* and *Staphylococcus epidermidus*, it was shown that biofilms of these organisms produced genetic variants [18], [19], [20], which were attributed to RecA-dependent recombination events. RecA is an important factor in DNA repair and furthermore the activator of the SOS response, which is a conserved pathway involved in DNA repair and restart of stalled replication forks [21], [22]. Previously it has been shown for *L. monocytogenes* that RecA contributes to survival of conditions mimicking the gastro-intestinal tract and translocation across the intestinal barrier [23]. Furthermore, *recA* and/or other SOS response genes of *L. monocytogenes* are activated during growth in a mouse macrophages cell line [24] and *in vivo* in mouse organs during infection [25]. Similarly, for *Escherichia coli* the SOS response appeared to be an essential mechanism during urinary tract infections [26], [27]. RecA and the SOS response are generally activated by events that result in the exposure of single stranded DNA, e.g. exposure to reactive oxygen species (ROS). Similarly, the production of variants in biofilms of the pathogen *Pseudomonas aeruginosa* grown in a drip-flow reactor was dependent on the presence and activity of RecA [28] and mutagenic repair of double stranded DNA breaks that occurred due to endogenous oxidative stress in these types of biofilms [29]. So far, nothing is known about the formation of genetic variants in *L. monocytogenes* biofilms and the possible mechanisms involved. Therefore, we aimed to investigate whether *L. monocytogenes* biofilms show mutagenesis and what mechanisms might be involved.

## Results

### Induced generation of variants in continuous-flow biofilms

**Figure 1:**
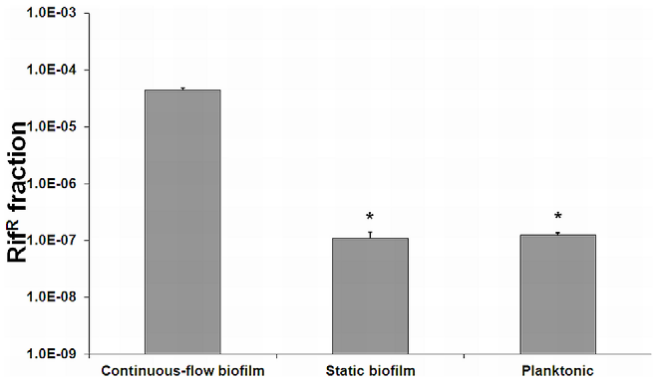
Induced generation of variants in continuous-flow biofilms. The graph presents the average and standard deviation of the rifampicin resistant fraction (0.05 µg/ml) of continuous-flow biofilms, static biofilms, and static planktonic cultures using three biological independent experiments. *Data points significantly different from the continuous-flow biofilms (p<0.05, *t*-test).

To investigate the formation of genetic variants in *L. monocytogenes* biofilms the occurrence of rifampicin-resistant variants in continuous-flow and static biofilms was investigated and compared with planktonic grown cultures. Resistance to the antibiotic rifampicin can be the result of point-mutations in the gene *rpoB* [30], and therefore the rifampicin-resistant fraction gives a good indication for the occurrence of genetic variants. The rifampicin-resistant fraction of continuous-flow biofilms was approximately 350-fold higher compared with planktonic cultures and approximately 400-fold higher compared with static biofilms (Fig. 1), while no significant difference between resistant fractions derived from static biofilms and planktonic cultures was observed. The results indicate that continuous-flow biofilms of *L. monocytogenes* show induced generation of genetic variants.

### Continuous-flow biofilms show radical-induced DNA damage

**Figure 2:**
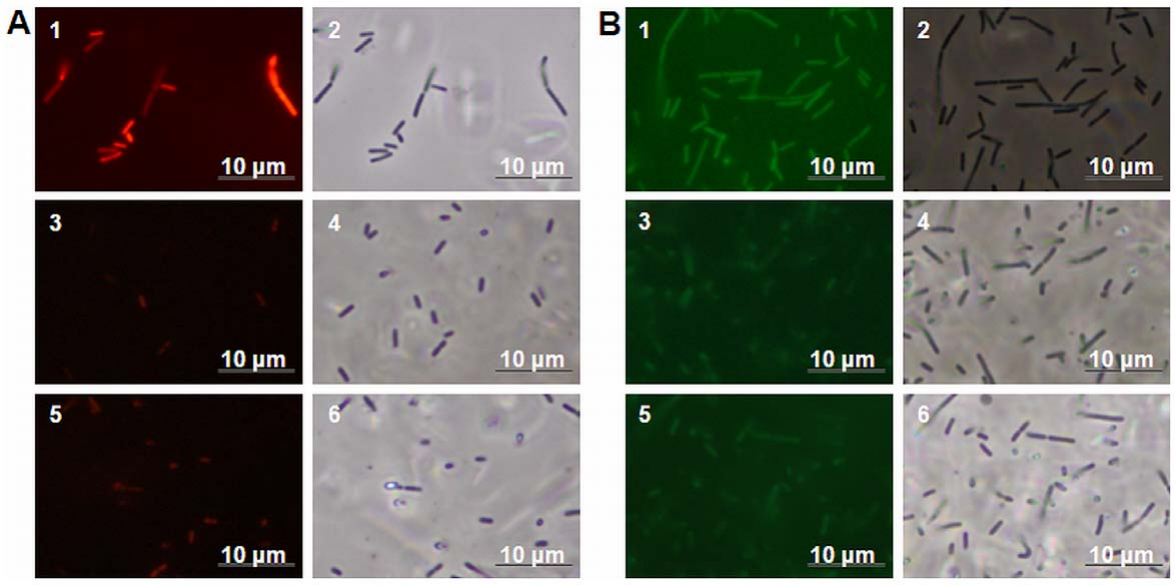
Induced radical formation during continuous-flow biofilm formation. The micrographs show fluorescence (1, 3, and 5) and phase contrast (2, 4, and 6) pictures of MitoSOX (A) and HPF (B) stained cells obtained from continuous-flow biofilms (1 and 2), static biofilms (3 and 4), and static planktonic cultures (5 and 6).

**Figure 3:**
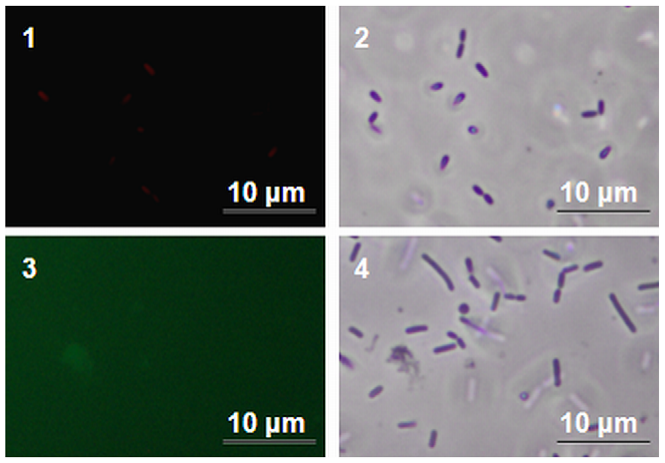
Radical inhibitors bipyridyl and thiourea inhibit radical formation during continuous-flow biofilm formation. Fluorescence (1 and 3) and phase contrast (2 and 4) pictures of MitoSOX (1 and 2) and HPF (3 and 4) stained cells obtained from continuous-flow biofilms grown in BHI with 0.05 mM bipyridyl and 50 mM thiourea.

**Figure 4:**
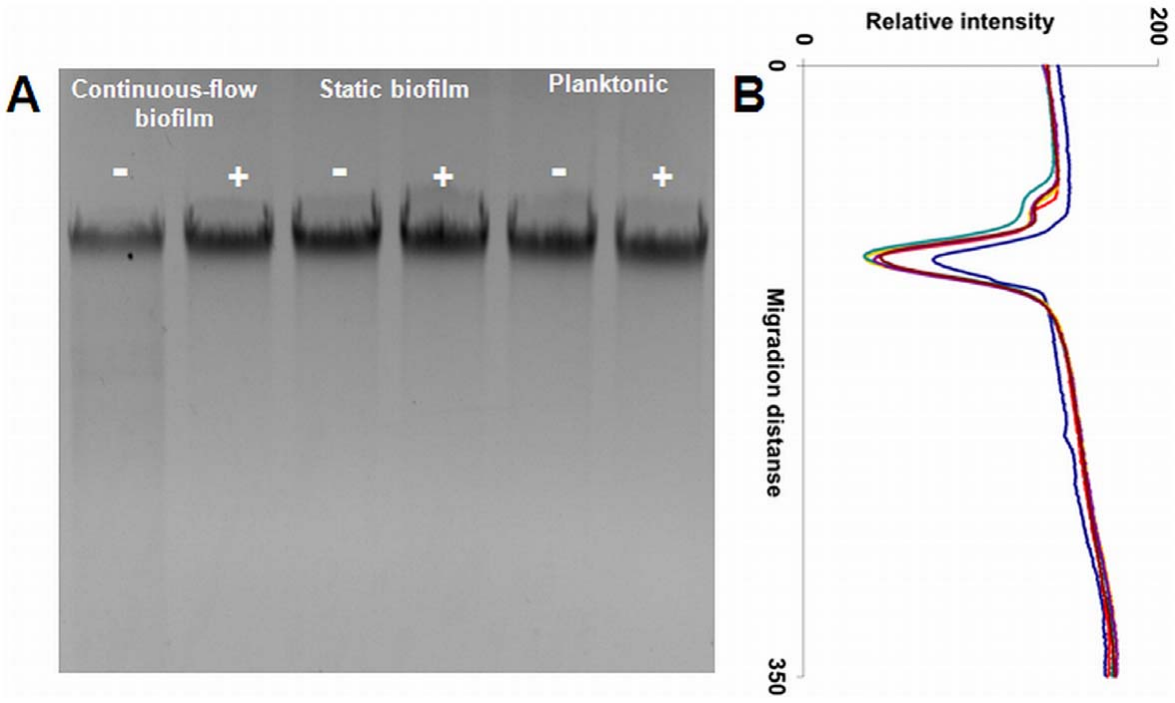
Radical formation during continuous-flow biofilm formation results in DNA damage. A) Ethidium bromide-stained agarose gel containing 0.5 µg genomic DNA isolated from cells obtained from continuous-flow biofilms, static biofilms, and planktonic cultures grown in BHI without (-) and with 0.05 mM bipyridyl and 50 mM thiourea (+). B) Relative intensity of each lane of the agarose gel plotted against the migration distance. DNA isolated from cells obtained from continuous-flow biofilms grown in BHI without (blue) and with bipyridyl and thiourea (red), static biofilms grown in BHI without (yellow) and with bipyridyl and thiourea (green), and static planktonic cultures grown in BHI without (purple) and with bipyridyl and thiourea (brown).

Since continuous-flow biofilms specifically show induced generation of variants, the question arises what the specific mechanism is behind this phenomenon. For *Pseudomonas aeruginosa* biofilms grown in a drip-flow reactor it was shown previously that the generation of variants was dependent on endogenous oxidative stress-mediated DNA damage and repair [29]. To investigate whether continuous-flow biofilms and not static biofilms or planktonic grown cells experienced oxidative stress, the formation of superoxide (Fig. 2A) and hydroxyl radicals (Fig. 2B) was investigated using the MitoSOX and HPF probes, respectively. Cells obtained from continuous-flow biofilms showed more intense fluorescence compared with that of cells obtained from static biofilms or planktonic cultures, which indicates that the formation of superoxide and hydroxyl radicals is specifically induced in continuous-flow biofilms. Furthermore, to verify that the fluorescence observed with the MitoSOX and HPF probes during continuous-flow biofilm formation was completely dependent on the generation of superoxide and hydroxyl radicals, the radical scavenger thiourea and iron chelator bipyridyl were added to the growth medium in concentrations that did not affect planktonic growth (results not shown). The addition of these radical inhibitors effectively prevented the formation of superoxide and hydroxyl radicals during continuous-flow biofilm formation (Fig. 3). To determine whether the chromosomal DNA of continuous-flow biofilm cells was affected as a result of the induced generation of radicals, chromosomal DNA was isolated from continuous-flow and static biofilm cells and planktonic grown cells grown in the presence and absence of the radical inhibitors bipyridyl and thiourea and inspected on an agarose gel (Fig. 4A). DNA isolated from continuous-flow biofilms grown in the absence of radical inhibitors showed increased deterioration compared with DNA isolated from continuous-flow biofilms grown in the presence of radical inhibitors, static biofilms, or planktonic cells. The DNA patterns were furthermore analyzed by densitometry measurements (Fig. 4B), which showed a lower peak for DNA isolated from continuous-flow biofilms grown in the absence of radical inhibitors and an increased signal for deteriorated DNA. These results indicate that continuous-flow biofilms and not static biofilms or planktonic cells experience increased radical formation and radical-induced DNA damage.

### RecA-mediated DNA repair is required for induced generation of variants in continuous-flow biofilms

**Figure 5:**
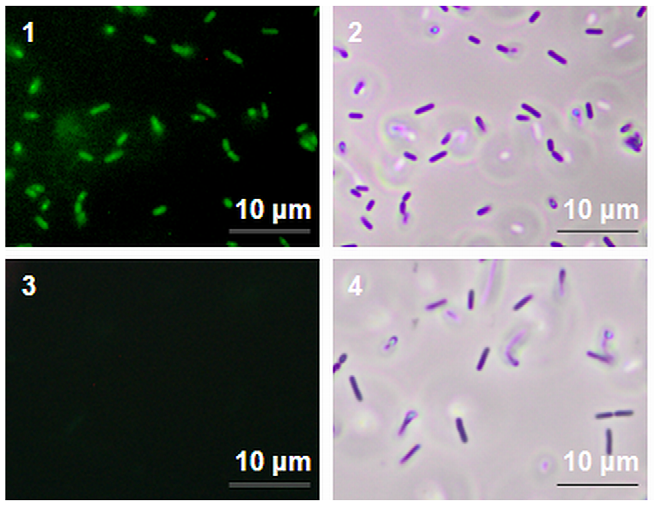
Activation of *recA* during continuous-flow biofilm formation is dependent on radical-induced DNA damage. Micrographs show fluorescence (1 and 3) and phase contrast (2 and 4) pictures of cells expressing EGFP from the *recA* promoter. Cells are obtained from continuous-flow biofilms grown in BHI without (1 and 2) and with (3 and 4) 0.05 mM bipyridyl and 50 mM thiourea.

**Figure 6:**
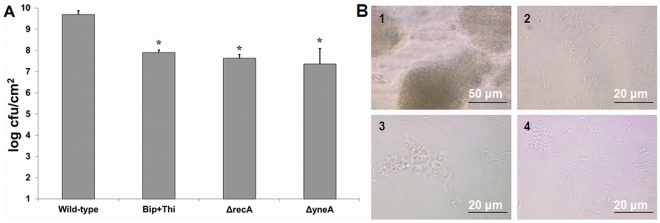
Continuous-flow biofilm formation is dependent on radical- and RecA-induced activation of *yneA*. Activation of SOS response member *yneA* is required to obtain fully grown continuous-flow biofilms that are composed of micro-colonies surrounded by knitted chains composed of elongated cells. A) The graph presents the average and standard deviation of the continuous-flow biofilm produced by the wild-type strain, the Δ*recA* mutant, and the Δ*yneA* mutant grown in BHI, and the wild-type strain grown in BHI with 0.05 mM bipyridyl and 50 mM thiourea using three biological independent experiments. *Data points significantly different from the wild-type strain grown in BHI (p<0.05, *t*-test). B) The micrographs present phase contrast pictures of continuous-flow biofilms of the wild-type strain grown in BHI without (1) and with 0.05 mM bipyridyl and 50 mM thiourea (2), and of the Δ*recA* mutant (3), and the Δ*yneA* mutant (4) grown in BHI.

**Figure 7:**
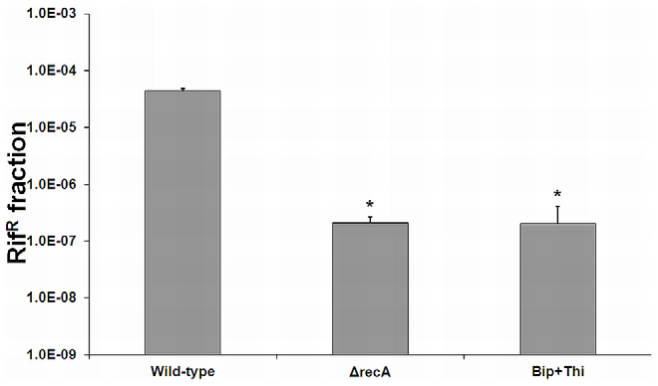
Generation of variants in continuous-flow biofilms is dependent on radical-induced DNA damage and RecA-mediated mutagenic DNA repair. The graph presents the average and standard deviation of the rifampicin resistant fraction (0.05 µg/ml) of continuous-flow biofilms of the wild-type strain and the Δ*recA* mutant grown in BHI, and the wild-type strain grown in BHI with 0.05 mM bipyridyl and 50 mM thiourea using three independent biological experiments. *Data points significantly different from the wild-type strain grown in BHI (p<0.05, *t*-test).

Previously, it has been shown that RecA is involved in the formation of variants in biofilms of several organisms. Furthermore, for *L. monocytogenes* RecA is also required for mutagenesis in planktonic cells [31] and after exposure to the mutagenic agent mitomycin C (Fig. S1). Also, *recA* is specifically activated in *L. monocytogenes* during continuous-flow biofilm formation and RecA-mediated activation of the SOS response member *yneA* is required to obtain fully-grown biofilms consisting of ball-shaped microcolonies surrounded by knitted-chains composed of elongated cells [7]. Using a *recA* promoter reporter we verified that *recA* is indeed activated in cells obtained from continuous-flow biofilms, but furthermore that activation of *recA* in continuous-flow biofilms is the result of radical-induced DNA damage (Fig. 5), since activation of *recA* was not observed in continuous-flow biofilms grown in the presence of radical inhibitors. Furthermore, consistent with previous results, Δ*recA* and Δ*yneA* deletion mutants showed a deficiency (100-fold) in continuous-flow biofilm formation due to the inability to form knitted-chains composed of elongated cells, which is dependent on the activation of SOS response factor *yneA* (Fig. 6). We now showed that the addition of radical inhibitors to the growth medium results in a similar marked reduction in continuous-flow biofilm formation due to the inability to activate *yneA* (Fig. 6A). While continuous-flow biofilms grown in the absence of the radical inhibitors showed microcolonies surrounded by a network of knitted-chains composed of elongated cells, continuous-flow biofilms grown in the presence of the radical inhibitors only showed very small microcolonies or single attached cells (Fig. 6B). These results indicate that for *L. monocytogenes* to obtain fully grown mature continuous-flow biofilms, radical-induced DNA damage is required to achieve RecA-mediated activation of the SOS response member *yneA*. Finally, to investigate the role of RecA and radical-induced DNA damage in the generation of variants in continuous-flow biofilms, the rifampicin-resistant fraction of continuous-flow biofilms was compared with the rifampicin-resistant fraction of the Δ*recA* mutant and the rifampicin-resistant fraction of continuous-flow biofilms grown in the presence of radical inhibitors (Fig. 7). The rifampicin-resistant fraction of continuous-flow biofilms grown in the absence of radical inhibitors was approximately 200-fold higher compared with Δ*recA* biofilms and biofilms grown in the presence of radical inhibitors. These results indicate that the increased generation of variants in *L. monocytogenes* continuous-flow biofilms is dependent radical-induced DNA damage and RecA-mediated mutagenic repair.

## Discussion

**Figure 8:**
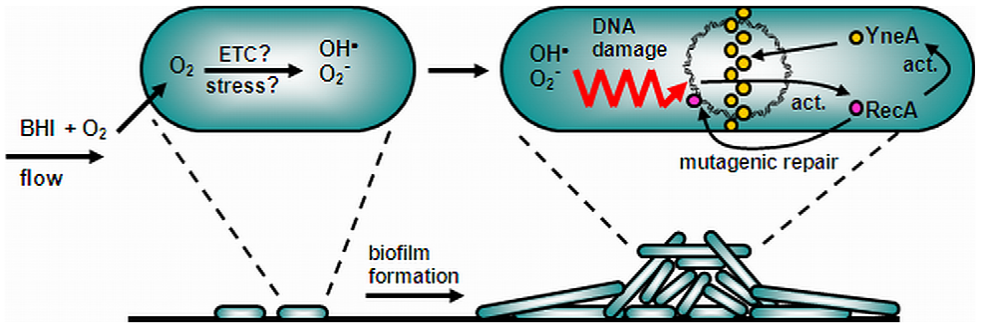
Proposed model for the generation of variants in *L. monocytogenes* continuous-flow biofilms. Continuous influx of oxygen saturated BHI in the flow-cells will result in relatively high oxygen concentrations in the attached cells. Intracellular oxygen is converted into superoxide and subsequently hydroxyl radicals. These ROS cause DNA damage and as result *recA* is activated. RecA subsequently mediates mutagenic repair of the damaged DNA, which results in the generation of genetic variants. Furthermore, activation of *recA* results in the activation of the SOS response and its regulon member *yneA*. YneA subsequently accumulates at the midcell to prevent septum formation, which results in cell elongation. Cell elongation is required to reach fully grown continuous-flow biofilms that consist of ball-shaped microcolonies surrounded by a network of knitted-chains composed of elongated cells.

Bacterial biofilms have been associated with the generation of genetic variants that subsequently might become persistent strains in the industry or human infections [15], [16], while the underlying mechanism of this phenomenon is largely unknown for most organisms. For *L. monocytogenes*, nothing is known thus far on the possible formation of genetic variants in biofilms and the underlying mechanisms. Our results now demonstrate that continuous-flow biofilms and not static biofilms show induced generation of genetic variants. While rifampicin resistant variants arose with a frequency of approximately 10^−7^ in planktonic cultures and static biofilms, which is similar to the frequencies reported previously for *L. monocytogenes*, *E. coli*, and *Streptococcus uberis* [32], [33], [34], continuous-flow biofilms showed a 350–400 fold induction in the rifampicin resistant fraction. The induced generation of rifampicin resistant variants in continuous-flow biofilms appears to be the result of oxidative DNA damage and RecA-mediated mutagenic repair of the damaged DNA (Fig. 8). These results are in line with previous observations on the role of RecA in the formation of genetic variants in biofilms of *S. pneumoniae*, *S. epidermidus*, and *P. aeruginosa* [18], [19], [20], [28]. Furthermore, a role for endogenous oxidative stress mediated DNA damage was indicated to be required for the formation of variants in *P. aeruginosa* biofilms [29], although the type of oxidants and their origin were not known in that study. In our study, using MitoSOX and HPF probes, we identified superoxide and hydroxyl radicals to be the specific types of oxidants that were produced in continuous-flow biofilms. These oxidants have been shown to cause serious damage to in particular DNA, proteins, and lipids [35], [36]. The production of ROS has furthermore been shown to be one of the primary host innate immune responses to invading microorganisms [37]. Our results might therefore also be indicative for the role of RecA and the SOS response in pathogenesis of *L. monocytogenes*.

Although we now identified the specific oxidants produced in continuous-flow biofilms that are associated with DNA damage and the induced generation of genetic variants, we do not yet know why the formation of these oxidants is increased during continuous-flow biofilm formation. It might be related with the continuous influx of oxygen saturated BHI broth in the flow-cells during continuous-flow biofilm formation. Notably, we observed a fivefold higher rifampicin-resistant fraction for planktonic cultures grown under shaking conditions compared with cultures grown under static conditions (Fig. S2), which is most likely related with a higher oxygen influx into the culture. Although *L. monocytogenes* is capable of growing under aerobic conditions, it is generally considered to be a microaerophilic and facultative anaerobic microorganism that performs best under reduced oxygen conditions [38]. This might be explained by the fact that *L. monocytogenes* contains an intact glycolysis and pentose phosphate pathway and a complete respiratory or electron transport chain (ETC), but an incomplete tricarboxylic acid (TCA) cycle because the α-ketoglutarate dehydrogenase is missing [39]. Continuous influx of oxygen in the flow-cells will result in relatively high intracellular oxygen concentrations, because oxygen is able to freely cross the cell membrane [40]. Intracellular oxygen by chance abstracts electrons that are leaking from intermediates of the ETC, which results in the formation of superoxide. Superoxide is able to target the iron-sulphur clusters in proteins, thereby releasing iron in the cytoplasm [41]. Free iron reacts with hydrogen peroxide that is produced by the dismutation of superoxide and forms the highly reactive hydroxyl radicals [36].

Alternatively, the increased formation of superoxide and hydroxyl radicals in continuous-flow biofilms might be related with the wide variety of different stresses that the bacterial cells experience in the different microniches of the biofilms [6]. For *L. monocytogenes* continuous-flow biofilms it was shown previously that expression of *sigB*, which encodes the activator of the class II stress response, and *hrcA* and *dnaK*, which encode the regulator and major chaperone of the class I heat-shock response, are highly induced [8], [9]. These results indicate that *L. monocytogenes* cells grown in continuous-flow biofilms experience stress. For several other organisms it has been shown that exposure to different stresses might result in oxidative stress on a molecular level. For instance, exposure of *E. coli* and *Staphylococcus aureus* to bactericidal antibiotics resulted in the generation of ROS due to perturbation of the ETC and subsequent leakage of electrons from ETC intermediates [42]. Similarly, exposure of *Bacillus cereus* cells to acid and heat resulted in the induction of radical formation, which corresponded with reduced viability [43], [44]. It is conceivable that the *L. monocytogenes* cells grown in continuous-flow biofilms experience stresses that might perturb the ETC, which subsequently results in the formation of superoxide and hydroxyl radicals.

Thus, although the underlying mechanism of the formation of ROS such as superoxide and hydroxyl radicals in continuous-flow biofilm formation remains to be elucidated, we have shown ROS to be a determinant of this type of biofilm formation and that this process is accompanied with a significant amount of DNA damage. Bacteria have evolved several mechanisms to cope with DNA damage, which include RecA and the SOS response (for reviews see [45], [46]). Activation of *recA* and the SOS response after DNA damage often results in the occurrence of genetic variants due to mutagenic DNA repair [21]. We now showed that RecA, besides its role in continuous-flow biofilm formation by activating SOS response factor *yneA* [7], is specifically involved in the induced generation of variants in continuous-flow biofilms of *L. monocytogenes* (Fig. 8), which was exemplified by the reduced generation of rifampicin resistant variants by the Δ*recA* mutant during continuous-flow biofilm formation. Our results further highlight the possible role of this type of biofilms in the development of resistance of *L. monocytogenes* against antibiotic therapies as a result of ROS-mediated DNA damage and RecA-mediated mutagenic repair. Similar observations were made for the occurrence of gentamycin resistant variants in *P. aeruginosa* biofilms [29]. However, in that study selection of gentamycin resistant variants was stimulated by growing the biofilms in the presence of a sublethal concentration of gentamycin. In addition, several studies using planktonic cells have previously shown that ROS formation and RecA/ SOS response-mediated mutagenic repair after exposure to sublethal levels of antibiotics could result in increased resistance against a range of antibiotics [47], [48]. Therefore, it has been postulated that novel therapeutic strategies should be focused on inhibition of mutations to combat bacterial antibiotic resistance [49]. Although most of the *L. monocytogenes* isolates are still susceptible to the majority of antibiotic therapies, increasing numbers of antibiotic resistant *L. monocytogenes* strains are being isolated around the globe from both clinical cases as well as foods and the food processing environment, including multi-drug resistant strains (reviewed in [50]). For instance, a recent study in France on the antimicrobial resistance of human isolates of *L. monocytogenes* between 1926 and 2007 showed a recent emergence of in particular tetracycline and ciprofloxacin resistant strains [51]. This study also showed that antibiotic resistance was the result of either acquirement of specific resistance genes or chromosomal mutations. Notably, a role for RecA and the SOS response in the acquirement of specific antibiotic resistance genes through integron activation and recombination has been shown for *E. coli* and *Vibrio cholera* [52], [53]. Furthermore, a study on the antimicrobial resistance of *L. monocytogenes* isolates from foods and the food processing environment showed that over 10% of the isolates displayed resistance to one or more antibiotics [54]. In our study, high levels of rifampicin resistant variants were detected in continuous-flow biofilms grown without exposure to sublethal levels of rifampicin, indicating that this type of bacterial biofilms might induce high frequency mutations and/or genetic recombination that could lead to generation of antibiotic resistant variants in natural systems or the food processing environment.

## Materials and Methods

### Strains, growth conditions, and biofilm formation

Single colonies of the strain *L. monocytogenes* EGD-e [39], its isogenic in-frame Δ*recA* deletion mutant [31], and its *recA* promoter reporter mutant EGD-e:PrecA-EGFP (enhanced GFP) [31] were used to grow overnight cultures (18 h) in brain heart infusion (BHI) broth (Becton Dickinson, Le Pont de Claix, France) at 20°C in 10 ml BHI broth in 50 ml polypropylene tubes (Greiner Bio-One, Frickenhausen, Germany). Static and continuous-flow biofilm formation experiments were performed as described in detail previously [8], [9]. In short, static biofilms were grown in 12-well polystyrene microtiter plates (Greiner Bio-One, Frickenhausen, Germany) containing 3 ml BHI broth, using a 1% inoculum of an overnight grown culture. After 48 h incubation at 20°C, the medium was removed and biofilms were washed three times with phosphate buffered saline (PBS; Merck, Darmstadt, Germany). Continuous-flow biofilms were grown for 48 h at 20°C in a flow cell (BST FC 281; Biosurface Technologies Corporation, Bozeman, USA). The flow cell was seeded using a diluted (1%) overnight grown culture that was left for 1 h to adhere after which BHI broth was pumped through with a flow of 10 ml/h.

### Detection of genetic variants

To detect the generation of genetic variants, approximately 2*10^9^ cells from the static and continuous-flow biofilms and planktonic cultures (grown for 24 h in BHI broth using a 1% inoculum of an overnight grown culture) were used for serial dilution in PBS and plated on BHI agar with and without 0.05 µg/ml rifampicin (Sigma-Aldrich, Steinheim, Germany). This rifampicin concentration is the MIC for *L. monocytogenes* EGD-e (results not shown). The plates were incubated for 2 days at 30°C and colonies were enumerated.

### Detection of radical formation

To detect radical formation in biofilm and planktonic cells, the reporter dyes MitoSOX (Molecular Probes, Invitrogen, USA), which is a derivative of hydroethidine, and 3′-(p-hydroxyphenyl) fluorescein (HPF; Invitrogen, Breda, The Netherlands) were used. MitoSOX binds to DNA after specific oxidation by superoxide and subsequently gives a red fluorescent signal [55]. HPF reacts with hydroxyl radicals and subsequently gives a green fluorescent signal [56]. Radical detection experiments were performed as described previously [43]. In short, biofilm or planktonic cells were resuspended in 1 ml PBS and MitoSOX (5 µM final concentration) or HPF (5 mM final concentration) were added. After incubation, cells were centrifuged (30 sec at 14000 x g) and pellets were dissolved in cold PBS. Cells were placed on microscope slides and analyzed by fluorescence microscopy.

### Microscopy

Phase contrast and fluorescence microscopy experiments were performed on a BX41 microscope (Olympus, Zoeterwoude, The Netherlands). Fluorescence of MitoSOX, HPF, and EGFP was visualized using the U-MWIG3 and MNIBA3 filters (Olympus, Zoeterwoude, The Netherlands) and images were acquired with a XC30 camera (Olympus, Zoeterwoude, The Netherlands) run by Olympus Cell^B^ software (Zoeterwoude, The Netherlands).

### Detection of genomic DNA integrity

Detection of the integrity of genomic DNA was performed as described previously [57]. In short, genomic DNA was isolated from biofilm and planktonic cells and 0.5 µg DNA was run on an agarose (Invitrogen, Breda, The Netherlands) gel (0.8 % w/v). Migration distance of genomic DNA was visualized by ethidium bromide (Bio-Rad, Veenendaal, The Netherlands) staining and densitometry analysis was performed using the Band Leader 3.0 analysis software.

### Statistical analyses

Significant differences in biofilm formation or the generation of variants were identified using levene's test for equality of variances and the independent-samples *t*-test (p<0.05) in SPSS.

## Supporting Information

Figure S1 **Induced generation of variants after mitomycin C exposure.** The graph presents the average and standard deviation of the rifampicin resistant fraction (0.05 µg/ml) of static planktonic cultures grown for 24 h at 20°C before (dark grey) and after exposure to 2 µg/ml mitomycin C for 1 h (white) or 2 h (light grey). Experiments were performed in three biological independent replicates. *Significantly different from the unexposed condition and the Δ*recA* mutant strain (p<0.05, *t*-test).
(TIF)

Figure S2 **Generation of variants in planktonic cultures.** The graph presents the average and standard deviation of the rifampicin resistant fraction (0.05 µg/ml) of planktonic cultures grown for 24 h at 20°C under static and shaking (180 rpm) conditions. Experiments were performed in three biological independent replicates. *Significantly different from the static condition (p<0.05, *t*-test).
(TIF)
