# Supplementary figures and images for: Generation of Variants in Listeria monocytogenes Continuous-Flow Biofilms Is Dependent on Radical-Induced DNA Damage and RecA-Mediated Repair
Source: PLoS One. 2011 Dec 5;6(12):e28590. doi: 10.1371/journal.pone.0028590 (PMC3230620; doi:10.1371/journal.pone.0028590)

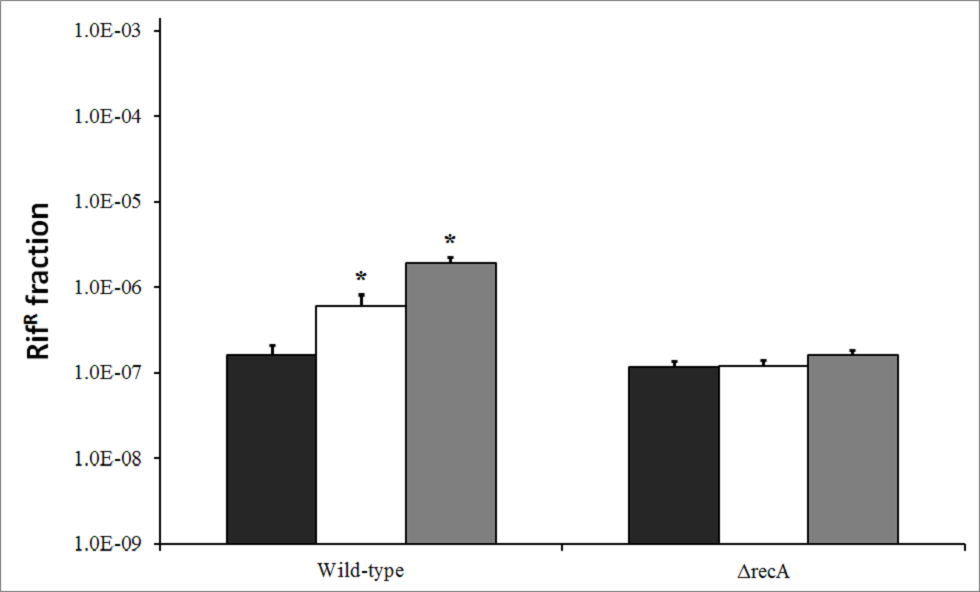

Supplement: Figure S1 — Induced generation of variants after mitomycin C exposure. The graph presents the average and standard deviation of the rifampicin resistant fraction (0.05 µg/ml) of static planktonic cultures grown for 24 h at 20°C before (dark grey) and after exposure to 2 µg/ml mitomycin C for 1 h (white) or 2 h (light grey). Experiments were performed in three biological independent replicates. *Significantly different from the unexposed condition and the ΔrecA mutant strain (p<0.05, t-test). (TIF) [file pone.0028590.s001.tif]

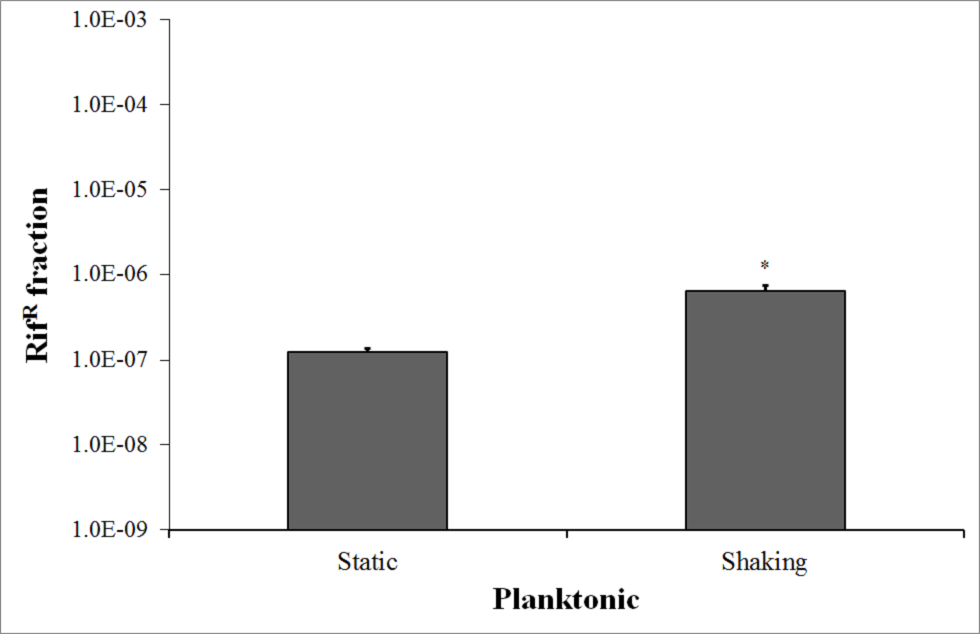

Supplement: Figure S2 — Generation of variants in planktonic cultures. The graph presents the average and standard deviation of the rifampicin resistant fraction (0.05 µg/ml) of planktonic cultures grown for 24 h at 20°C under static and shaking (180 rpm) conditions. Experiments were performed in three biological independent replicates. *Significantly different from the static condition (p<0.05, t-test). (TIF) [file pone.0028590.s002.tif]
